# Evaluating risk factors of radiation pneumonitis after stereotactic body radiation therapy in lung tumor: Meta-analysis of 9 observational studies

**DOI:** 10.1371/journal.pone.0208637

**Published:** 2018-12-06

**Authors:** Chi Lu, Zhang Lei, Hongbin Wu, Hongda Lu

**Affiliations:** 1 Department of Oncology, The Central Hospital of Wuhan, Tongji Medical College, Huazhong University of Science and Technology, Wuhan, P.R. China; 2 Cancer Research Institute of Wuhan, Wuhan, Hubei, P.R. China; European Institute of Oncology, ITALY

## Abstract

**Background:**

In this study, we assessed the association of SBRT (stereotactic body radiotherapy) dose and volume with radiation pneumonitis (RP) risk in lung tumor.

**Methods:**

Relevant articles were identified up to April 2018, using following databases; Medline, EMBASE, Cochrane Library, and China National Knowledge Infrastructure (CNKI). The pooled OR (odds ratio) with 95% CI (confidence interval) data [mean ± SD (standard deviation)] obtained from different studies was analyzed by statistical analysis using a fixed-effects model or a random-effects model when appropriate.

**Results:**

The analysis was based on nine observational studies, which were identified based on the study selection criteria. Between RP and non-RP patients, no difference was observed based on age, but significant differences were observed based on planning target volume (PTV), mean ipsilateral lung dose (MLD), total MLD, and V5, V10, V20 and V40 (the percentage of lung volume exceeding 5, 10, 20 and 40 Gy). In addition, PTV >145 cm^3^, total MLD ≥4.7 Gy, V5 ≥26.8%, V10 >12% and V20 ≥5.8 were associated with RP risk. Overall, the grade assessments of V5 and V20 revealed moderate quality evidence.

**Conclusion:**

The present study indicated V5 and V20 as major risk factors for RP after SBRT treatment in lung tumor. In addition, it was observed that lung DVH (Dose Volume Histogram) patterns should be assessed more carefully, while predicting RP incidence after SBRT.

## Introduction

Stereotactic body radiation therapy (SBRT) has been an important treatment option for certain cancers, which was first reported by Blomgren H *et*. *al*. in 1995 [1]. Furthermore, it has been widely used in extracranial tumors [2]. Recently, SBRT has also become a standard treatment option for inoperable early-stage non-small cell lung cancer (NSCLC) [3] patients. It has shown a local control rate of up to 97% after two years, and a survival rate of up to 64% after three years in NSCLC patients [4]. However, SBRT has also shown a potential risk of radiation pneumonitis (RP), similar to conventional radiotherapy. RP has been categorized as one of the most general toxicities of SBRT, with an incidence rate of more than 50%, and the percentage of symptomatic RP (grade≥2) ranges within 9%-28% [5–16]. Furthermore, grade 2 RP symptoms are observed most of the time, which appear to be fatal in patients due to inoperable NSCLC, and is usually combined with additional medical issues. RP has also been observed to cause certain chronic complications, such as pulmonary fibrosis and pulmonary insufficiency, which subsequently limits quality of life, along with treatment failure [17].

SBRT consists of unique fractions and dose distribution, which are very different from conventional radiotherapy. There are even some differences between these two methods in terms of RP incidence. In recent years, some studies have attempted to predict risk factors associated with RP after SBRT treatment. However, the results have been inconclusive. For instance, some studies identified the important relationship of planning target volume (PTV) and mean ipsilateral lung dose (MLD) with RP, while other studies did not observe these associations [5,6,8,10,11,13]. These variable results can be attributed to methodological problems and small number of cases, which probably led to conflicting results. Therefore, in the present study, we attempted to evaluate the risk factors of RP with specific focus on dose and volume of SBRT through meta-analysis with an intent of potentially developing effective therapeutic approach.

## Methods

### Search strategy

Relevant articles were searched until April 2018 from five databases: Medline, EMBASE, Cochrane Library and China National Knowledge Infrastructure (CNKI). The following key words were used: (((((SBRT) OR Radiosurgery) OR stereotactic body radiotherapy)) AND ((((radiation pneumonitis) OR radiation pneumonia)) OR lung toxicity)) AND ((Lung cancer) OR Lung tumor). Human studies in the English and Chinese language were selected. In addition, the reference lists from these identified studies were further searched for relevant reviews and articles.

### Study selection

The following criteria were used to select studies for the meta-analysis: (1) randomized, case–control and cohort studies; (2) studies that specifically diagnosed patients for primary lung cancer and pulmonary metastases by biopsy; (3) studies that confirmed the induction of RP through laboratory or radiologic examinations after SBRT; (4) studies with sufficient data to analyze dose-volume factors. Studies were excluded when the patients underwent re-irradiation or did not have sufficient data. All disagreements were resolved by consensus among the investigators.

### Data collection and analysis

The data obtained from relevant articles were independently collected by two reviewers. The following information were extracted: year of publication, author’s name, number of patients, treatment plan, and dose-volume parameters.

### Statistical analysis

The meta-analysis was performed according to the recommendations of the Cochrane Collaboration, with the use of Review Manager Software version 5.2 and the Grading of Recommendation Assessment, Development and Evaluation (GRADE) system[18]. The data (mean ± standard deviation [SD]) on age, PTV average value, MLD average value, total MLD average value, V5 average value, V10 average value, V20 average value, and V40 average value from RP and N-RP groups was, obtained from different studies and assessed. The pooled data (odds ratio [OR] and 95% confidence interval [CI]) of different factors was used to calculate logOR and standard error [SE], along with its assessment as risk factors. Optimal cut-off values were used for different factors. In addition, the heterogeneity among studies was evaluated using the *I*^*2*^-test and *P*-value. When the *I*^*2*^-value was ≤50% and the *P-*value was >0.1, the fixed effect model was used for the meta-analysis. However, when the *I*^*2*^-value was >50% (significant heterogeneity) and the *P*-value was ≤0.1, the random effect model was used. In order to confirm the stability of these studies, sensitivity analyses were performed by omitting one study at a time. Overall, a *P*-value of <0.05 was considered statistically significant.

## Results

### Description of studies

**Fig 1:**
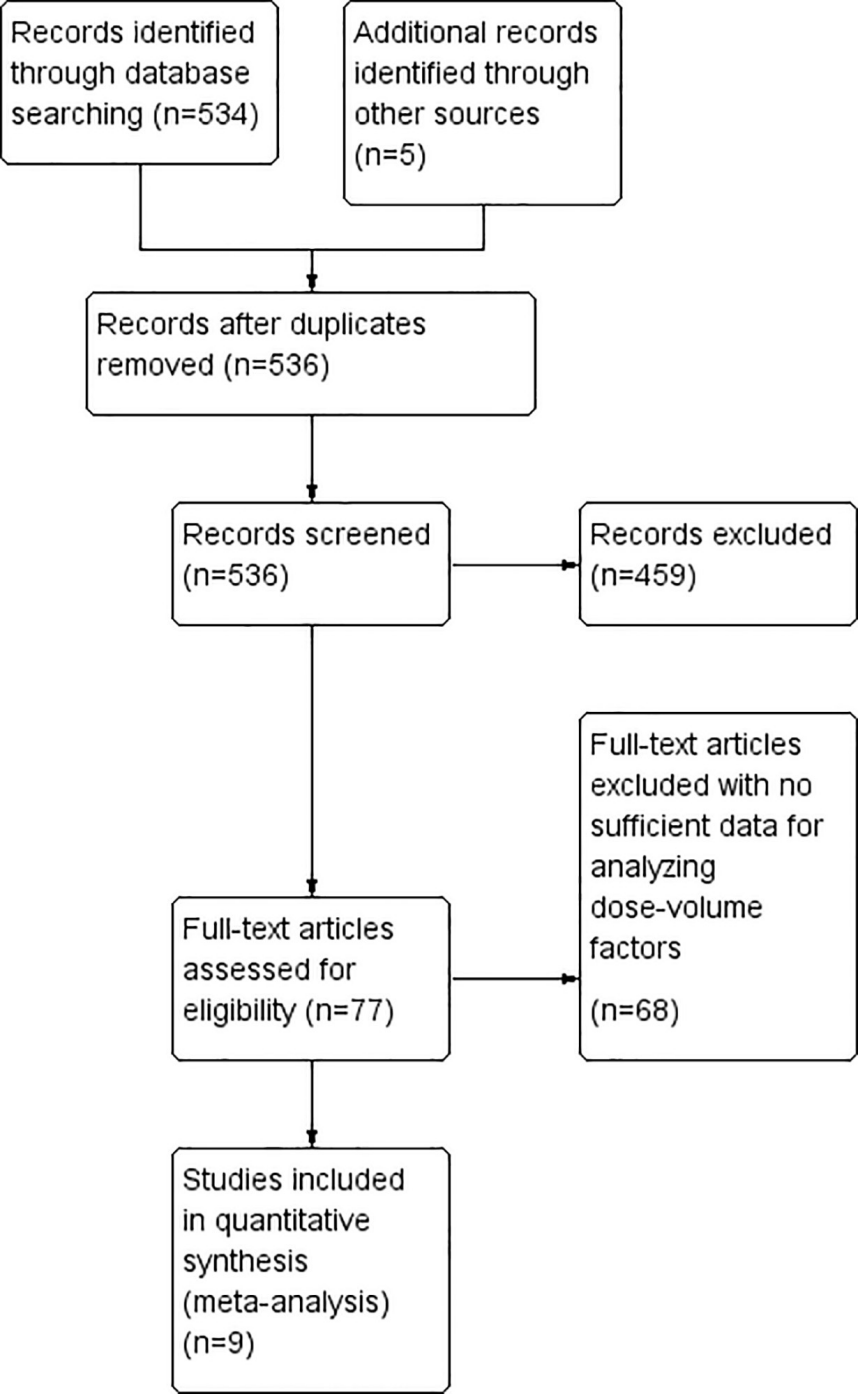
PRISMA flow chart depicting the study selection process.

The complete study selection process is outlined in Fig 1. Initial search led to the identification of 534 relevant studies. Subsequently, five additional studies were selected by cross-checking the references. Among these, 459 studies were excluded based on the information not consistent with the selection criteria after reviewing their titles, key words and abstract. In addition, 68 studies were further excluded due to insufficient data for dose-volume factors. Finally, nine studies that met all study selection criteria were selected for the meta-analysis.

These observational studies in English language were published between the years 1995 and 2018. All the included observational studies described the diagnosis criteria of RP, RP scale and its dose-volume parameters. The study characteristics of all these studies are presented in Table 1.

**Table 1:** Characteristics of the included studies.

Study	Pt or lesions	MedianAge (year)	Dose	Median PTV (cc)	Median follow-up (months)	RP ≥grade 2 (no.)	Dose-volume factors for RP after SBRT
Yamashita 2007[13]	25	77	48Gy/4-6Fr	43.9	17	7	CI
Ricardi 2009[12]	63	71.7	45Gy/3Fr or 26Gy/1Fr	NA	30.9	9	Ipsilateral MLD
Guckenberger 2010[8]	59	67	37.5Gy/3Fr or 26Gy/1Fr	33	13	11	Total MLD, Ipsilateral MLD, V2.5–50
Barriger 2012[6]	143	74	24-66Gy/3-5Fr	48.3	17	15	Total MLD, V20
Matsuo 2012[9]	74	77	48Gy/4Fr	32.5	31.4	15	PTV, V20,V25
Aibe 2013[5]	30	80	50Gy/3-5Fr	27.5	36.5	3	GTV
Bongers 2013[7]	79	75.5	54-60Gy/3-12Fr	149.4	13	8	Contralateral MLD, ITV
Moré 2014[10]	20	68	34-60Gy/1-5Fr	55.36	6	5	NA
Nakamura 2016[11]	56	78	48-56Gy/4Fr	23.8	12.5	6	PTV, GTV, Total MLD, V5-V50

MLD: mean lung dose; NA: not available; Pt: patients

### Qualitative analysis

**Fig 2:**
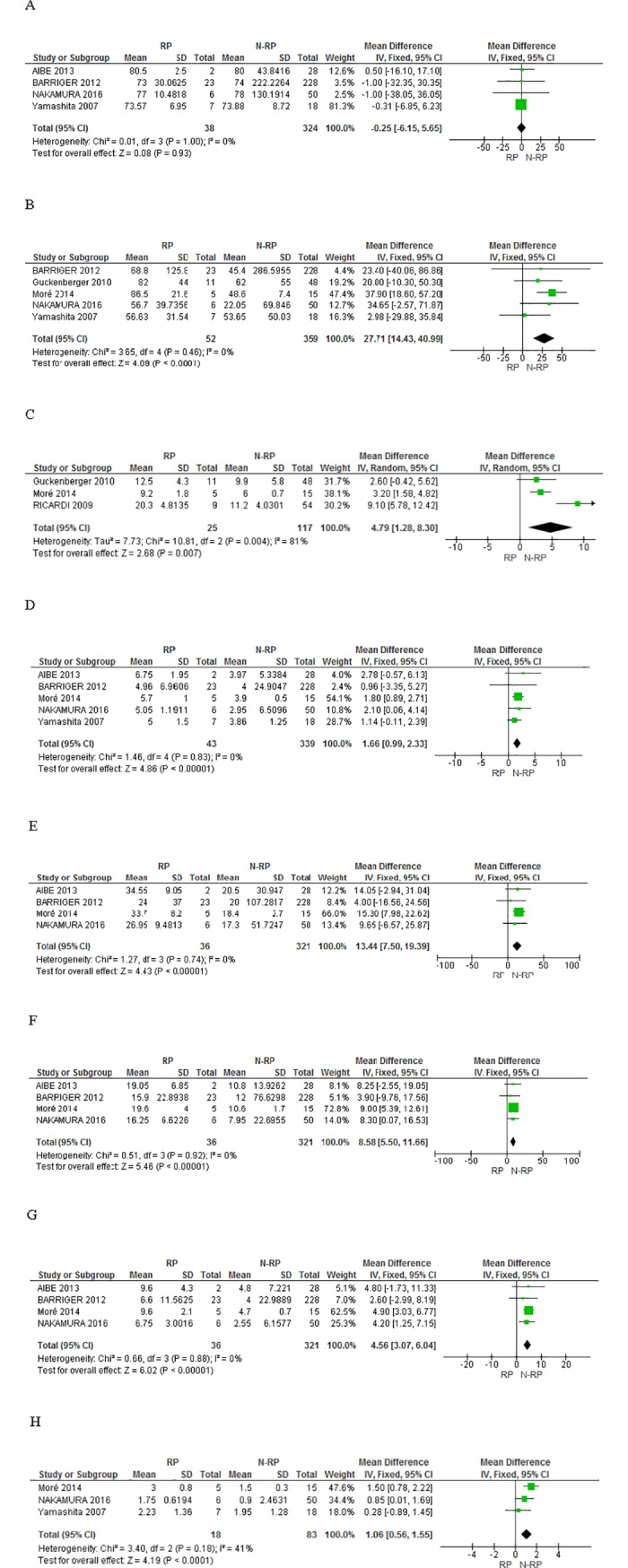
Forest plots representing the association between clinical, dose-volume factors and risk of RP ≥grade 2. The comparison between RP and non-RP patients is shown based on the following factors: age (panel A), PTV average value (panel B), MLD average value (panel C), total MLD average value (panel D), V5 average value (panel E), V10 average value (panel F), V20 average value (panel G), V40 average value (panel H).

**Fig 3:**
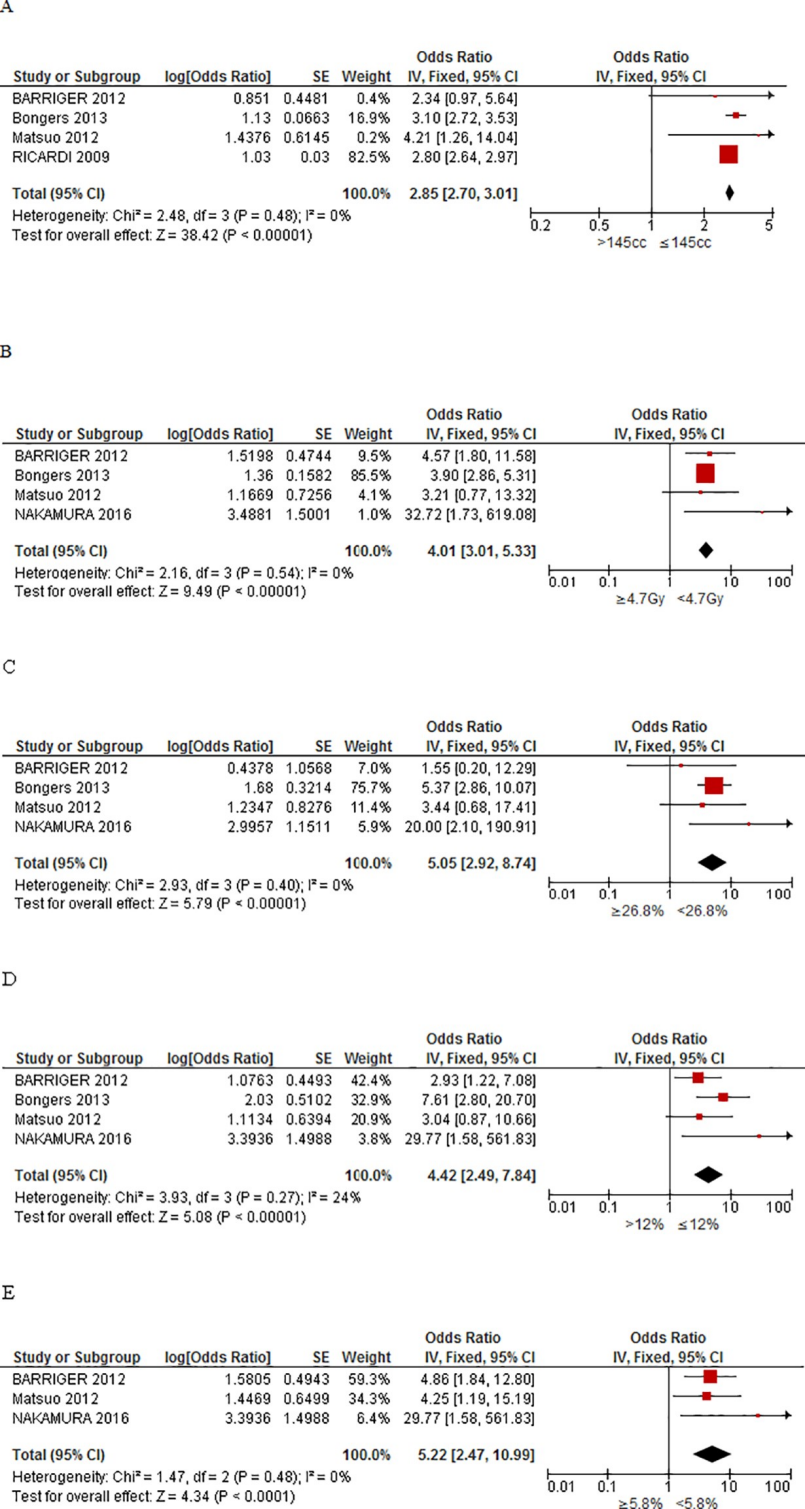
Forest plots representing the association between optimal cut-off values and risk risk of RP ≥grade 2. The optimal cut-off values are shown: PTV>145cc (panel A), total MLD ≥4.70 Gy (panel B), V5 ≥26.80% (panel C), V10 >12% (panel D), and V20 ≥5.80% (panel E).

A total of 13 relevant factors for RP ≥grade 2 were investigated from these nine studies, including the patient’s age, PTV, MLD, total MLD, and V5, V10, V20 and V40 (the percentage of lung volume exceeding 5, 10, 20 and 40 Gy). The outcomes of the dosimetric parameters for RP grade ≥2 were reported by the dichotomous and continuous data in all studies. The complete meta-analysis results of various factors between RP and non-RP patients are presented in Fig 2 (panels A-H). There was no difference in age between the RP and non-RP patient populations (MD = -0.25; 95% CI = -6.15, 5.65; *P* = 0.93; panel A). However, the meta-analysis results revealed a significantly higher value for PTV, MLD and total MLD (panels B, C and D) in RP patients, when compared to non-RP patients (MD = 27.71, 95% CI = 14.43, 40.99, *P*<0.0001; MD = 4.79, 95% CI = 1.28, 8.3, *P* = 0.007; MD = 1.66, 95% CI = 0.99, 2.33, *P*<0.00001; respectively). Due to high heterogeneity in MLD, the analysis was performed based on the random effect model. Furthermore, significant differences in V5, V10, V20 and V40 values (panels E, F, G and H) were also observed between RP and non-RP patients (MD = 13.44, 95% CI = 7.5, 19.39, *P*<0.00001; MD = 8.58, 95% CI = 5.5, 11.66, *P*<0.00001; MD = 4.56, 95% CI = 3.07, 6.04, *P*<0.00001; MD = 1.06, 95% CI = 0.56, 1.55, *P*<0.0001). In addition, important predictive factors for RP ≥grade 2 after SBRT were analyzed based on following optimal cut-off values: PTV >145 cm^3^, total MLD ≥4.7 Gy, V5 ≥26.8%, V10 >12% and V20 ≥5.8. These cut-off values were different in the original articles, so we choose the smallest of these values as optimal cut-off point. All these exhibited an association with RP risk (OR = 2.85, 95% CI = 2.7, 3.01, *P*<0.00001; OR = 4.01, 95% CI = 3.01, 5.33, *P*<0.00001; OR = 5.05, 95% CI = 2.92, 8.74, *P*<0.00001; OR = 4.42, 95% CI = 2.49, 7.84, *P*<0.00001; OR = 5.22, 95% CI = 2.47, 10.99, *P*<0.0001; respectively; Fig 3 panels A, B, C,D and E). All data are summarized in Table 2. The Effect Estimates indicate Mean Different or Odds Radio according to the statistical methods shown in Table 2.

**Table 2:** Summary of the meta-analysis depicting the association between dosimetric factors and risk of RP≥ grade 2 after SBRT in lung cancer.

Dose-volume parameters	Studies (*n*)	Statistical method	Heterogeneity		Effect Estimate
			*P*	*I*^*2*^ (%)	
Age	4	Mean Difference (IV, Fixed, 95% CI)	1.00	0	-0.25 [-6.15, 5.65]
PTV	5	Mean Difference (IV, Fixed, 95% CI)	0.46	0	27.71 [14.43, 40.99]
MLD	3	Mean Difference (IV, Fixed, 95% CI)	0.004	81	4.79 [1.28, 8.30]
Total MLD	5	Mean Difference (IV, Fixed, 95% CI)	0.83	0	1.66 [0.99, 2.33]
V5	4	Mean Difference (IV, Fixed, 95% CI)	0.74	0	13.44 [7.50, 19.39]
V10	4	Mean Difference (IV, Fixed, 95% CI)	0.92	0	8.58 [5.50, 11.66]
V20	4	Mean Difference (IV, Fixed, 95% CI)	0.88	0	4.56 [3.07, 6.04]
V40	3	Mean Difference (IV, Fixed, 95% CI)	0.18	41	1.06 [0.56, 1.55]
PTV: ≤145 cc *vs*. >145 cc	4	Odds Ratio (IV, Fixed, 95% CI)	0.48	0	2.85 [2.70, 3.01]
Total MLD: <4.70 Gy *vs*. ≥4.70 Gy	4	Odds Ratio (IV, Fixed, 95% CI)	0.54	0	4.01 [3.01, 5.33]
V5: <26.80% *vs*. ≥26.80%	4	Odds Ratio (IV, Fixed, 95% CI)	0.4	0	5.05 [2.92, 8.74]
V10: ≤12% *vs*. >12%	4	Odds Ratio (IV, Fixed, 95% CI)	0.27	24	4.42 [2.49, 7.84]
V20: <5.80% *vs*. ≥5.80%	3	Odds Ratio (IV, Fixed, 95% CI)	0.48	0	5.22 [2.47, 10.99]

### Grading the strength of the evidence

The nine studies included in the present analysis were non-randomized. Thus, the initial grade was low-quality based on the GRADE approach. The MLD subgroup was further downgraded by one level due to inconsistency and high heterogeneity. The imprecision of effect estimate and indirect evidence was not applied in the present analysis. When the magnitude of the specific effect was large (OR>2 or OR>5), the study grade was upgraded by one or two levels, respectively. These observational studies usually provide true and accurate effect estimates. However, due to the observational nature of these analyzed studies, the dose-response gradient could not be actually assessed. Based on the evidence, the grade assessment of V5 and V20 subgroups were of moderate quality, while other subgroups showed low quality data. All GRADE evidences are summarized in S1 Table.

### Sensitivity analysis

The sensitivity analysis was performed by omitting one study at a time to assess their impact on changes in the overall results. Since all studies involved subjective judgments, the sensitivity analysis was attempted to obtain some measure of the stability and reliability of the overall results. Interestingly, based on the sensitivity analysis, no significant differences were observed between groups. In addition, an attempt to assess for publication bias was also performed through funnel plot analysis. However, due to the very few studies for each parameter, this could not be performed.

## Discussion

The present meta-analysis study analyzed the dose-volume parameters that could impact the incidence of RP in lung tumor patients after SBRT treatment. These results revealed that the following parameters were the obvious risk factors: PTV, MLD, total MLD, V5, V10, V20 and V40. However, patient age had no correlation with the incidence of RP. The study conducted by Bledsoe TJ *et al*. revealed that during conventional lung radiotherapy, the dose parameters could be used as RP predictors, and its risk could be reduced by constraining these lung tissue dose-volumes [19]. However, although RP is the most common complication after SBRT treatment in lung tumor [17,20,21], determining how to constrain these proposed dose-volume parameters remains unclear. In this context, a study conducted by Barriger *et al*. revealed that total lung MLD and V20 were associated with RP. In their study, 23 of 251 patients (9.2%) had 2–4 grade RP [6]. When MLD was ≤4 Gy, 4.30% of these patients developed grade 2 RP, while when MLD was >4 Gy, 17.60% of these patients developed grade 2–4 RP. However, factors such as PTV, V5 and V10 were not associated with RP. Another study conducted by Bongers *et al*. revealed that total MLD, internal target volume (ITV), V5 and V10 were RP indicators [7]. Furthermore, in another retrospective analysis of 59 patients conducted by Guckenberger *et al*., PTV, total MLD, MLD and V2.5-V50 were observed to be associated with RP [8]. In contrast, the study conducted by Matsuo *et al*. indicated that only PTV, V20 and V25 were important predictors of RP, while factors including MLD, V5, V10, V15, V30 and V40 were not correlated with the development of RP [9]. All these studies indicate variable correlations between dose-volume parameters and RP incidence. Especially, age was found to be a risk factor for RP in most studies. According to one hypothesis, this may be attributed to worse performance by old people. However, our results showed that patient median age was not associated with higher rate of RP. This inconsistency may partially be due to 1) limited studies (4 observational studies) with relavant data, and 2) no consistency between age and lung function.

In pooled analysis by Nan Bi’, 31 eligible studies on SBRT displayed RP as most frequent complication (grade ≥3) in 2% of the patients, but no data on its risk factors [22]. However, our data was consistent with pooled analysis by Jing Zhao, wherein they showed lung V20 and MLD significantly affecting RP [23]. But this study also had no specific dosimetric constraints about RP risk factors and assessment of evidence quality. Interestingly, our meta-analysis demonstrated that when dosimetric parameters met the following specific constraints (i.e. PTV >145 cm^3^, total MLD ≥4.7 Gy, V5 ≥26.8%, V10 >12% and V20 ≥5.8), the incidence of RP was quite high. Moreover, PTV, MLD, total MLD, and V5, V10, V20 and V40 dose volume factors were significantly different between RP and non-RP patients. Since these dose-volume factors depend on each other, the shape of the dose volume histogram (DVH) should be more significant than the point dose on this DVH curve in predicting the rate of RP incidence. Importantly, low quality evidence about grade assessment of age, PTV, MLD, total MLD and V10, and moderate quality evidence about V5 and V20 subgroups grade assessment indicate that additional research is required to accurately assess the impact of each specific factor. Since among these above-mentioned dose-volume parameters, a grade assessment of V5 and V20 reflected moderate quality evidence, so they can serve as major factors for RP incidence.

In addition, it is important to highlight that despite few studies (3–5) included in our analysis and among them one study being much larger than others, they all align on the same line in most of the forest plots. The sensitivity analysis also established that no specific study significantly affected the meta-analysis estimate. Therefore, one can say that important limitation of having few studies with unequal sample size did not impact the overall results and even the observed heterogeneity was also low.

The schedules of radiotherapy used in our study ranged from 26 Gy/1 fraction to 60 Gy/12 fractions, and were particular important for late responding tissues like lungs (and late event like pneumonitis). There is an ample amount of data in the literature about lung injury after conventionally fractionated radiotherapy, while with stereotactic radiotherapy very less information is out there. There are multiple radiobiological mathematical models for calculating the dose in different radiotherapy schedules, but none of them are uniformly validated and approved. Also, the prescription dose do not always correlate with dose distribution in normal lung for highly conformal SBRT, and it rely more on target volume, location and conformity. Thus, different schedules of radiotherapy may not correlate with RP in SBRT. This view is also shared and supported by some previous studies focusing on different locations of lung tumor [24–26].

The National Comprehensive Cancer Network (NCCN) guidelines described the maximum dose constraints for SBRT in lung cancer, but there was no information on dose-volume constraints [27]. Based on the results obtained from the present study, it is evident that the reasonable shape of lung DVH can better reduce the incidence of RP, when compared to the maximum dose. More specifically, further consideration should be given to V5 and V20 parameters obtained from the lung DVH.

Finally, there were still some limitations in the present meta-analysis. First, some studies did not have sufficient data, and were thereby excluded. Second, few studies described all risk factors, and the number of samples were also not large enough. Third, the risk of RP after SBRT is undoubtedly correlated with many factors, and among these we only analyzed dosimetric data and age as potential variables. However many other factors like tobacco smoking, baseline lung function, comorbidity and concomitant systemic therapy were not analyzed. Due to the limited information provided in the published literature, we could not analyze all possible factors and provide accurate recommendations. Fourth, the observational nature of these studies could also introduce risk of bias in the results. Thus, the results of the present meta-analysis should be interpreted with caution, and warrants additional future studies.

## Conclusion

The present meta-analysis provides clear evidence on the correlation among dose-volume factors, including PTV, MLD, total MLD, V5, V10, V20 and V40, which are risk factors for the incidence of RP after SBRT treatment in lung tumor. Furthermore, the present study reinforces the fact that a specific lung DVH pattern is significant for predicting RP incidence, while V5 and V20 factors have the highest potential to predict RP incidence after SBRT treatment.

## Supporting information

S1 Checklist PRISMA 2009 checklist in this meta-analysis. (DOC)

S1 Table All GRADE evidences. (DOC)
