# Supplementary material for: Evaluating risk factors of radiation pneumonitis after stereotactic body radiation therapy in lung tumor: Meta-analysis of 9 observational studies
Source: PLoS One. 2018 Dec 6;13(12):e0208637. doi: 10.1371/journal.pone.0208637 (PMC6283643; doi:10.1371/journal.pone.0208637)
Supplement: S1 Table — (DOC) [file pone.0208637.s002.doc]

**Supplementary Table 1:** GRADE assessment of the quality of evidence

| **Quality assessment** | | | | | | | **Effect** | | **Quality** |
| --- | --- | --- | --- | --- | --- | --- | --- | --- | --- |
|
| **No of studies** | **Design** | **Risk of bias** | **Inconsistency** | **Indirectness** | **Imprecision** | **Other considerations** | **Relative (95% CI)** | **Absolute** |  |
| **Age (Better indicated by lower values)** | | | | | | | | | |
| 4 | Observational studies^1^ | Serious^1^ | No serious inconsistency | No serious indirectness | No serious imprecision | None | - | MD 0.25 lower (6.15 lower to 5.65 higher) |  VERY LOW |
| **PTV (Better indicated by lower values)** | | | | | | | | | |
| 5 | Observational studies^1^ | Serious^1^ | No serious inconsistency | No serious indirectness | No serious imprecision | None | - | MD 27.71 higher (14.43 to 40.99 higher) |  VERY LOW |
| **Ipsilateral MLD (Better indicated by lower values)** | | | | | | | | | |
| 3 | Observational studies^1^ | Serious^1^ | Serious^2^ | No serious indirectness | No serious imprecision | None | - | MD 4.79 higher (1.28 to 8.3 higher) |  VERY LOW |
| **Total MLD (Better indicated by lower values)** | | | | | | | | | |
| 5 | Observational studies^1^ | Serious^1^ | No serious inconsistency | No serious indirectness | No serious imprecision | None | - | MD 1.66 higher (0.99 to 2.33 higher) |  VERY LOW |
| **V5 (Better indicated by lower values)** | | | | | | | | | |
| 4 | Observational studies^1^ | Serious^1^ | No serious inconsistency | No serious indirectness | No serious imprecision | None | - | MD 13.44 higher (7.5 to 19.39 higher) |  VERY LOW |
| **V10 (Better indicated by lower values)** | | | | | | | | | |
| 4 | Observational studies^1^ | Serious^1^ | No serious inconsistency | No serious indirectness | No serious imprecision | None | - | MD 8.58 higher (5.5 to 11.66 higher) |  VERY LOW |
| **V20 (Better indicated by lower values)** | | | | | | | | | |
| 4 | Observational studies^1^ | Serious^1^ | No serious inconsistency | No serious indirectness | No serious imprecision | None | - | MD 4.56 higher (3.07 to 6.04 higher) |  VERY LOW |
| **v40 (Better indicated by lower values)** | | | | | | | | | |
| 3 | Observational studies^1^ | Serious^1^ | No serious inconsistency | No serious indirectness | No serious imprecision | None | - | MD 1.06 higher (0.56 to 1.55 higher) |  VERY LOW |
| **PTV ≤145 cc** | | | | | | | | | |
| 4 | Observational studies^1^ | Serious^1^ | No serious inconsistency | No serious indirectness | No serious imprecision | Strong association^3^ | OR 2.85 (2.7 to 3.01) |  |  LOW |
| **MLD <4.70 Gy** | | | | | | | | | |
| 4 | Observational studies^1^ | Serious^1^ | No serious inconsistency | No serious indirectness | No serious imprecision | Strong association^3^ | OR 4.79 (1.28 to 8.3) |  |  LOW |
| **V5 <26.80%** | | | | | | | | | |
| 4 | Observational studies^1^ | Serious^1^ | No serious inconsistency | No serious indirectness | No serious imprecision | Very strong association^4^ | OR 5.05 (2.92 to 8.74) |  |  MODERATE |
| **V10 ≤12%** | | | | | | | | | |
| 4 | Observational studies^1^ | Serious^1^ | No serious inconsistency | No serious indirectness | No serious imprecision | Strong association^3^ | OR 4.42 (2.49 to 7.84) |  |  LOW |
| **V20 <5.80%** | | | | | | | | | |
| 3 | Observational studies^1^ | Serious^1^ | No serious inconsistency | No serious indirectness | No serious imprecision | Very strong association^4^ | OR 5.22 (2.47 to 10.99) |  |  MODERATE |

^1^ Case-control
^2^ There is heterogeneity
^3^ The magnitude of the exposure effect is relatively large
^4^ The magnitude of the exposure effect is very large
